# Concrete and Abstract Concepts in Primary Progressive Aphasia and Alzheimer’s Disease: A Scoping Review

**DOI:** 10.3390/brainsci13050765

**Published:** 2023-05-05

**Authors:** Martina Mancano, Costanza Papagno

**Affiliations:** 1 Center for Mind/Brain Sciences, University of Trento, 38068 Rovereto, Italy; martina.mancano@unitn.it; 2 CISMed Interdepartmental Center for Medical Sciences, University of Trento, 38122 Trento, Italy

**Keywords:** svPPA, semantic dementia, Alzheimer’s disease, concreteness effect, semantic categories, concrete concepts, abstract concepts, anterior temporal lobe

## Abstract

The concreteness effect (CE), namely a better performance with concrete compared to abstract concepts, is a constant feature in healthy people, and it usually increases in persons with aphasia (PWA). However, a reversal of the CE has been reported in patients affected by the semantic variant of Primary Progressive Aphasia (svPPA), a neurodegenerative disease characterized by anterior temporal lobe (ATL) atrophy. The present scoping review aims at identifying the extent of evidence regarding the abstract/concrete contrast in Alzheimer’s disease (AD) and svPPA and associated brain atrophy. Five online databases were searched up to January 2023 to identify papers where both concrete and abstract concepts were investigated. Thirty-one papers were selected and showed that while in patients with AD, concrete words were better processes than abstract ones, in most svPPA patients, there was a reversal of the CE, with five studies correlating the size of this effect with ATL atrophy. Furthermore, the reversal of CE was associated with category-specific impairments (living things) and with a selective deficit of social words. Future work is needed to disentangle the role of specific portions of the ATL in concept representation.

## 1. Introduction

Concepts (and words) can be classified as concrete or abstract, the difference being that the first concepts are “material” objects that are tangible and can be experienced through our senses (e.g., car, bear), while abstract concepts are not (e.g., happiness, courage). Psycholinguistic studies on healthy participants [1,2] demonstrated that concrete items are processed faster than abstract ones, and neuropsychological studies [3] have shown that in patients with aphasia due to an anterior lesion involving the left inferior frontal gyrus, therefore, in persons with nonfluent aphasia, this advantage, called concreteness effect (CE), is magnified. However, a subgroup of patients presents with an inversion of this effect, the so-called reversal of CE (see [4] for a review). In general, these patients have bilateral but asymmetric lesions in the anterior part of the temporal lobes, with the left side more damaged than the right. Indeed, most patients described in the literature had suffered from herpes simplex encephalitis [5] or were patients in the early stages of the semantic variant of Primary Progressive Aphasia (svPPA), a subtype of the broader spectrum of Frontotemporal dementia.

Frontotemporal dementia (FTD) is a neurodegenerative disease characterized by temporal and frontal lobar atrophy. It presents with a variety of symptoms, which allows distinguishing three main subtypes [6]: the behavioral variant of FTD (bvFTD), with primarily behavioral and executive symptoms [7], a nonfluent Primary Progressive Aphasia (nfPPA), with primarily language symptoms, and semantic dementia (SD). The latter can start with a predominant left atrophy, which produces a semantic variant of PPA (svPPA). SvPPA’s main symptom is the degradation of semantic memory, with difficulties in name retrieval and loss of semantic features of objects. Gorno-Tempini et al. [8] identified a third form of PPA that they named logopenic (lPPA); lPPA more often represents an atypical onset of Alzheimer’s disease (AD). In fact, AD patients also present with early language and semantic deficits [9,10].

BvFTD also can show linguistic deficits, including difficulties with abstract concepts [11,12]. Therefore, while a reversal of the CE has been found in some patients with SD, in bvFTD, the CE seems to increase.

It must be acknowledged, however, that tests for semantic memory mainly focus on concrete concepts (e.g., the Pyramid and Palm Trees test, [13]), whereas fewer investigate the contrast between concrete and abstract concepts.

Different theories have been proposed to account for this dissociation, none of which can explain the reversal of the CE as they provide a quantitative difference either in terms of the number of available representations, both verbal and sensory–perceptual for concrete concepts and only verbal for abstract ones [14], or in terms of larger contextual support for concrete words [15] or in terms of the number of attributes that would be higher in the case of concrete words/concepts than in the case of abstract ones [16,17]. To overcome the quantitative explanation, Crutch and Warrington [18] proposed a difference in the organization: concrete concepts organized in categories and abstract ones relying on associations with other items, with a different meaning depending on the context. However, abstract concepts can also be referred to as categories [19]. Indeed, emotional words have already been considered as categories [20], as social words [21] and quantity-related concepts [22].

In contrast to the lack of a satisfactory theoretical explanation, what can be taken for granted is the presence of different anatomical correlates for the two types of items. However, there is no agreement on which these correlates are. In people with aphasia (PWA), an increase in the CE has been associated with vascular damage in the territory of the left middle cerebral artery that involves the prefrontal cortex, while, as already reported, it appears that the large majority of cases with a reversed CE suffered an anterior temporal lesion, generally bilateral but more evident on the left side, as it has been confirmed in studies on patients after left or right temporal pole resection [23], and on patients submitted to direct electrical stimulation during awake surgery [24].

Unfortunately, neuroimaging data are not totally in line with the clinical evidence. Indeed, while apparently the role of the left inferior frontal gyrus (IFG) for abstract words is undoubtedly established [25], a recent meta-analysis [26] confirmed that concrete and abstract words processing involves at least partially segregated brain areas and that the inferior frontal gyrus is crucial for abstract words, but also demonstrated that more posterior temporoparietal-occipital regions seem to be crucial for processing concrete words. The lack of consistency between neuropsychological and neuroimaging data might be explained by the different populations tested, usually young people in fMRI experiments vs. old people in neuropsychological samples. Another reason could be the frequent overlap of the terms and abstract/concrete with high-/low-imageability. Concreteness means the extent to which a word refers to a tangible item, whereas imageability refers to the extent to which an item can evoke a mental image [27]. It is well-known that the two features operate differently on naming and recall [28,29,30,31] and cannot be considered, therefore, interchangeable.

Another topic of debate is the fact that the reversal of the CE was initially described in single case reports [32,33,34,35], prompting the “negationist” researchers to suggest that these people were simply outliers with high education. However, subsequent group studies [36,37,38,39,40] confirmed the existence of this effect, especially in patients affected by svPPA, who were contrasted with people affected by bvFTD, who showed, in turn, an increased CE. Nonetheless, Jefferies et al. [41]’s group study found that svPPA patients were more impaired in abstract compared to concrete concepts and rejected the hypothesis that a reversal of CE is a hallmark of these patients. They further supported these findings with a neurostimulation study [42], where they showed that inhibitory transcranial magnetic stimulation (TMS) targeting anterior temporal regions particularly impaired performance with low-imageability rather than with high-imageability items. On the other hand, no one considered the reversal of the CE a constant feature of the disease, but the point is that when it occurs, it is mainly in patients with svPPA.

To summarize, both a CE and its reversal exist. It is not clear whether damage to the anterior temporal poles, as found in svPPA, has a role in producing selective damage to concrete concepts. To shed light on the debate concerning the role of the anterior temporal poles in concrete word processing and the occurrence of the reversal of the CE in svPPA, we analyzed the published cases of neurodegenerative patients in which language was studied with a specific focus on abstract and concrete dissociation to verify whether there is a sharp distinction based on the presence of atrophy in specific regions of the brain. Therefore, we conducted a scoping review to identify and map the available evidence and clarify which is the volume of the literature on this topic, namely the dissociation between abstract and concrete concepts/words and associated brain atrophy.

## 2. Materials and Methods

We followed a previously developed protocol to create and report scoping reviews, using the Preferred Reporting Items for Systematic Reviews and Meta-analyses extension for Scoping Reviews (PRISMA-ScR) Checklist [43].

### 2.1. Eligibility Criteria

Title, abstract, and full-text articles were screened for eligibility based on the inclusion criteria: (1) both concrete and abstract concepts were investigated within the same group/patients, (2) tested patients with a probable diagnosis of either AD or Primary Progressive Aphasia (the three types: namely svPPA, nfPPA, lPPA), and/or bvFTD, (3) original studies, (4) written in English, and (5) peer-reviewed. Papers including only abstract or only concrete (and not both) categories were excluded; we considered only papers where both abstract and concrete concepts were investigated and compared. We included both group studies and single-case reports.

### 2.2. Information Sources and Search

The following online bibliographic databases were searched up to January 2023: MEDLINE (accessed by PubMed, https://www.ncbi.nlm.nih.gov/pubmed (accessed on 18 January 2023)), PsycARTICLES (via EBSCOHost, https://search.ebscohost.com (accessed on 19 January 2023)), PsycINFO (via EBSCOHost), Scopus (https://www.scopus.com (accessed on 20 January 2023), accessed via University of Trento), and Web of Science (https://webofknowledge.com/ (accessed on 20 January 2023)). For one included paper [44], we contacted the first author to obtain the full text.

Search keywords were the following: (1) “dementia”, “semantic dementia”, “FTD”, “Alzheimer” AND (2) “concreteness”, “abstract concepts”, “concrete concepts”, “concrete words”, and “abstract words”.

The references were exported into a text format and uploaded on Rayyan software [45].

On Rayyan, we removed duplicates after careful detection.

To prevent the risk of bias, both authors carried out autonomously (i.e., with Rayyan ‘blind on’ modality) title and abstract screening. After both authors completed the screening, resulting conflicts were reviewed and resolved by discussion and consensus. After that, both authors proceeded to the full-text screening of the potentially relevant papers, which were included in accordance with the abovementioned inclusion criteria.

Relevant data were extracted from the included papers by one reviewer, while the other verified the accuracy of the data.

We abstracted data related to participants’ features (sample size, presence of a control group, diagnosis, atrophy extension), methods (tasks, type, and the number of stimuli, semantic and grammatical categories investigated), and outcomes (behavioral results and correlation between atrophy site and behavioral results).

We grouped the records according to the diagnosis of patients included in the study (only PPA, only AD, both PPA and AD). We summarized the results considering more specifically the contrast between concrete and abstract concepts performance, and, when present, the correlation between imaging and behavioral results, the distinction between different grammatical classes (i.e., nouns, verbs, adjectives), and different categories of concrete (living/non-living) and abstract (social, emotion, etc.) words.

The administered tasks varied, including synonym judgement task, picture naming, naming to definition, elicited speech by means of the Cookie Theft picture description task [46], autobiographical memory, and semantic priming paradigms.

When considering the contrast between concrete and abstract domains, we collapsed results together across comprehension, production, and priming paradigms.

## 3. Results

**Figure 1:**
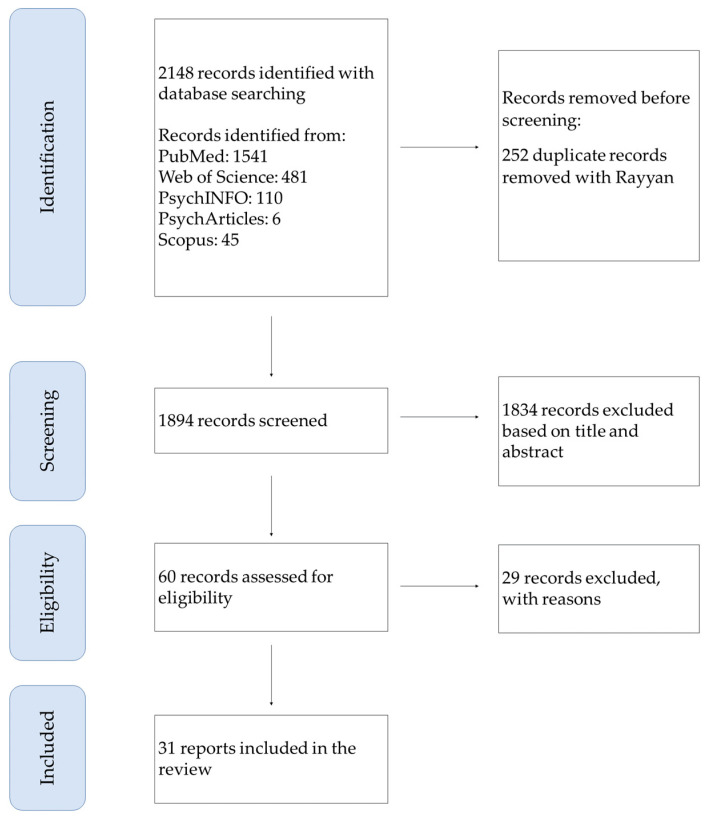
Flow diagram of study selection and inclusion.

The literature search identified 2148 articles, including 1541 from PubMed, 481 from Web of Science, 110 from PsychInfo, six from PsychArticles, and 45 from Scopus. Of these, 252 were removed as duplicates. After the title, abstract screening, and full-text articles assessment, 31 papers survived the final selection and were included in the analysis. Figure 1 shows the search and selection process.

Among the 31 included records, 19 were experimental studies investigating semantic representation in Primary Progressive Aphasia patients. Three of them were case reports, two were case series, and the remaining were all group studies.

Among the group studies, six records investigated semantic representations in AD patients, while the other six were group studies investigating semantic representations in both PPA and AD patients.

For reasons of coherence, we reported all studies referring to “Semantic Dementia” and “svPPA” under one label, “svPPA”.

Results are shown in Tables S1–S3.

### 3.1. Synthesis of Results

#### 3.1.1. Participants

PPA records included 163 bvFTD and 492 PPA patients: 63 nfPPA, 113 lPPA, 40 unclassified, and 276 svPPA. AD records included 214 patients.

#### 3.1.2. Contrast Concrete/Abstract

In this subsection, we present the results of the contrast between concrete and abstract concepts without distinguishing for grammatical class (e.g., nouns, verbs) and categories (e.g., animals, emotions). The results based on these variables are discussed below.

- Semantic variant Primary Progressive Aphasia (svPPA)

Out of the 24 studies on svPPA patients, 14 (58.3%, 147 patients) showed a better performance with abstract compared to concrete concepts ([20,33,34,35,36,37,38,39,40,47,48,49,50,51]. Six studies (25%, 99 patients) found better performance with concrete compared to abstract concepts [22,41,44,52,53,54]. Finally, four studies (16.6%, 30 patients) found no significant difference in performance between abstract and concrete concepts [55,56,57,58].

- Behavioral variant Frontotemporal Dementia (BvFTD)

Three studies (66 patients) found better performance with concrete than abstract concepts in bvFTD patients [36,37,56]. Two studies (82 patients) found a similar performance between the two domains [39,58].

- Logopenic Primary Progressive Aphasia (lPPA)

Both studies, which included lPPA patients, found better performance with concrete compared to abstract concepts [44,56].

- Nonfluent Primary Progressive Aphasia (nfPPA)

The three studies, which included nfPPA patients, found better performance with concrete compared to abstract concepts [39,44,56].

- Alzheimer’s disease (AD)

Out of the 12 studies on AD patients, five (41.6%, 106 patients) reported better performance with concrete compared to abstract concepts [50,59,60,61,62], while four studies (33.33%, 58 patients) found no difference [20,51,57,58]. Two studies (16.66%, 39 patients) reported opposite results, with a better performance with abstract compared to concrete concepts [63,64]. The remaining study was unclassifiable [65]. In this study, the authors did not contrast concrete and abstract concepts, although they tested both.

Results are summarized in Table 1.

#### 3.1.3. Site of Atrophy in PPA

Among the included records, only a portion of PPA studies (*n* = 13) reported imaging data of patients’ atrophy ([33,34,35,36,37,38,39,40,44,52,54,55,58], while another study reported the correlation between svPPA patients’ performance and atrophy sites [20].

With regards to the svPPA patients, the majority of these studies reported atrophy in the Anterior Temporal Lobe (ATL), bilaterally [38,40,55], bilaterally with a predominance on the left side [33,35,36,39,40,44,52,56,58], limited to the left side [34]. The specific atrophic regions of the ATL were the inferolateral [35,40], medial temporal cortex [34], bilateral Inferior Temporal Gyrus (ITG) and left Fusiform Gyrus (FG) [37], medial and lateral [38], inferior [44].

A minority of them also reported significant atrophy in some frontal regions, such as the left Inferior Frontal Gyrus (IFG) and the orbitofrontal cortex, particularly on the left side [39]. Atrophy of the insula was also reported [36,38].

With regards to the bvFTD patients, the reported sites of atrophy were frontal [58], frontal lobe and modest right temporal lobe atrophy [36], bilateral IFG, orbitofrontal cortex, superior temporal gyrus [37], and bilateral frontal and temporal lobes [39].

Two studies reported sites of atrophy for nfPPA: left middle frontal, inferior temporal, and middle temporal regions [39], and asymmetric frontal atrophy [44].

Stockbridge et al. [44] also reported left temporoparietal atrophy for their lPPA cohort.

There were no data regarding atrophy in AD patients.

#### 3.1.4. Correlation between Atrophy and Reversal of CE

Some of these studies analyzed the relationship between the svPPA patients’ semantic performance and cortical atrophy. Five group studies reported a positive correlation between the size of reversal of CE (better performance with abstract compared to concrete concepts) and atrophy in anterior temporal regions: right anterolateral temporal cortex [40], left anterior temporal cortex [36], parahippocampal gyrus and portions of left ATL [37], left ATL, medial and lateral [20], right ventral and left superior temporal regions [38], and left ATL [39].

In [37], the authors also observed that decreased abstractness of speech in bvFTD was related to atrophy in the left IFG, left superior frontal gyrus, left anterior cingulate, and bilateral caudate.

#### 3.1.5. Different Semantic Effects across Grammatical Classes: Nouns, Verbs, Adjectives

- Nouns

Seven studies assessed semantic performance on nouns [22,34,35,36,37,38,39].

In their svPPA case report, Breedin et al. [34] tested the patient’s performance on different categories of concrete nouns and on concrete contrasted to abstract nouns. In the different categories of concrete nouns, the patient revealed a better performance with inanimate nouns compared to biological ones; on picture naming, verbal fluency on phonemic cues and word-definition tasks, the patient performed better with abstract than with concrete nouns.

In his longitudinal svPPA case report, Macoir [35] reported a better performance on similarity judgement for the abstract compared to the concrete meaning of homophones in a semantic similarity task in the first testing session, which disappeared in the following two with the progression of the disease.

Cousins et al. [36], in a similar way, found higher accuracy in svPPA patients for abstract noun triads compared to concrete ones in a similarity judgement task, whereas bvFTD patients demonstrated the opposite pattern, and controls showed no effect of concreteness.

Production was also investigated: Cousins et al. [37,38] and Cho et al. [39] used the Cookie Theft Picture description task [46], and the elicited descriptions were transcribed; in particular, they measured the abstractness of produced nouns and found that svPPA patients produced significantly more abstract nouns than bvFTD patients, and the degree of abstractness of produced nouns positively correlated with other measures of semantic impairment [37,38]. Cousins et al. [38] also observed a longitudinal decrease in the concreteness of produced nouns only in the first group, so there was a positive relationship between the duration of disease and abstractness of speech. Similarly, in Cho et al. [39], svPPA, compared to bvFTD, nfPPA and controls, produced more abstract nouns.

In a lexical decision task with pairs of different categories of concrete and abstract nouns [22], semantic priming was observed for all categories in controls, while it was abolished in svPPA patients only for one abstract category of pairs, namely social pairs.

- Verbs and Nouns

Seven studies investigated the dissociation in the semantic representation of nouns and verbs across different tasks [33,34,47,50,52,53,60].

One study found no difference between performance on nouns and verbs [53]. The authors found a similar increased CE across nouns and verbs triplets in svPPA patients compared to controls in a synonym matching task, with a better comprehension of more imageable than less imageable items regardless of their grammatical class. In a verb-picture naming task, svPPA, lPPA, and unclassified PPA patients all showed a CE for verbs, with increased concreteness related to better performance [44]. Instead, Breedin et al. [33], in a synonym judgement task where the patient was asked to choose the less related word, found better performance with verbs than with noun triplets. However, in a following test, they investigated three types of verb triplets: non-relational triplets (distractor verb opposite in meaning to the probe), manner triplets (distractor verb that expresses the same action but executed in a different way), and relational triplets (distractor verb expresses the same event but assigns thematic roles differently). The patient’s performance was significantly impaired only with manner verbs, for which the sensorimotor (or concrete) component is relevant.

Other studies found different effects of concreteness depending on the word class of the stimuli. In a study on semantic priming in AD patients, Bushell and Martin [60] found different priming effects depending on the word class and concreteness: neither controls nor AD showed priming for abstract nouns and non-motion verbs, but controls showed priming for both motion verbs and concrete nouns, and AD patients only for concrete nouns.

Yi et al. [50], using a naming-to-description task with concrete and abstract nouns, and motion and cognitive verbs, found a CE for nouns in AD patients but not for verbs and a reversal of CE in svPPA patients, with better performance on cognition compared to motion verbs, but no difference in performance between abstract and concrete nouns.

Similarly, in a concreteness judgement task including nouns and verbs, in which patients are asked whether the stimulus is concrete or abstract, svPPA patients were prone to misclassify long concrete words as abstract, but this effect was apparent only with verbs [47]. Bonner et al. [40] included only verbs and found that svPPA patients were better at similarity judgement of abstract compared to concrete verbs (reversal of CE), opposite to controls’ performance.

This reversal CE was not replicated by Catricalà et al. [52] on svPPA patients using the same tasks as [40,50]; instead, they found no significant difference between abstract and concrete verbs.

Papagno et al. [34] also did not find any CE (or its reversal) for verbs in a svPPA patient: in synonymy and word-definition tasks, the patient was indistinguishable from controls with verbs; however, the same patient was impaired in concrete but not in abstract nouns in those tasks, i.e., the reversal of CE was specific for nouns.

- Adjectives

No effect of concreteness with adjectives was found by [34], as well as in [55], who found a similar performance across concreteness levels, with no significant difference between concrete (colour, dimension, physical property) and abstract (human propensity, value) performance in synonymy judgement and adjective-to-noun matching tasks.

Other studies did not provide information regarding the grammatical class of the stimuli used.

#### 3.1.6. Abstract and Concrete Categories

Beyond the dichotomous abstract vs. concrete concepts distinction, twelve studies investigated the dissociation between different categories within concrete, abstract, or both concrete and abstract domains [20,22,33,34,35,51,54,55,58,62,63,64]. Two studies also investigated emotion as a dimension rather than a category [62,63].

- Concrete domain: the distinction between living and non-living entities

Five studies investigated the distinction between living and non-living concepts processing within the concrete category [22,33,34,35,51].

Breedin et al. [33] found, in addition to the worse performance with concrete nouns, a worse performance in answering questions on perceptual compared to non-perceptual features and a better performance with non-living (tools) than with living (animals) concepts in a synonymy judgement task.

In [35]’s longitudinal svPPA case report, instead, there was no difference in performance between living and non-living items across a variety of different tasks (semantic similarity judgement, word-to-picture matching, word definition, word spelling to dictation, picture naming and naming to definition). Differently from both, Papagno et al. [34] showed a selective loss of conceptual knowledge of living entities but with better preserved visual features than functional/associative ones.

This dissociation was not confirmed in a subsequent study [51], in which AD and svPPA patients consistently produced a similar performance in living and non-living in a picture naming task. A dissociation was instead observed in naming by description, where AD performed better on inanimate entities than biological ones. In a following svPPA case, Catricalà et al. [22] tested for priming effects across several concrete (animals, tools) and abstract categories. They observed an hyperpriming effect (increased priming) only for animal (living) word pairs.

- Abstract domain: the role of emotion and social concepts

Nine studies investigated emotion and/or social abstract concept categories [3,20,22,34,51,54,58,63,64]. Two studies also considered emotion as a dimension [62,63].

Giffard et al. [62] tested four types of semantic priming, manipulating the word concreteness and the type of relationship, that could be either emotional (negative) or neutral: concrete neutral (table-chair), concrete emotional (viper-cobra), abstract neutral (motive-reason), and abstract emotional (grief-sadness). AD patients showed a CE only in the neutral concrete and abstract priming conditions, whereas in the emotional conditions, they showed equivalent priming for concrete and abstract words: the authors suggested that emotion could be one of the main components that bind semantically close concepts together in AD. Martin and Fedio [63] asked AD patients and controls to read aloud words belonging to four categories and select which of four drawing best represented it (symbol referent test); they found that patients were impaired in objects (e.g., ‘chair’), actions (e.g., ‘sit’), and modifiers (e.g., ‘strong’), but not in emotion (e.g., ‘love’) words.

They also asked patients to provide pleasantness ratings for neutral, positive (pleasant) and negative (unpleasant) words and found they did not differ from controls in this task.

The role of emotion has been further confirmed [64], with AD patients performing better on immediate recall of emotional abstract (positive and negative valence, e.g., friend, hate) than a neutral concrete word (e.g., thermometer) lists and, moreover, better with negative than with positive words.

Hsieh et al. [58] tested AD, svPPA, and bvFTD patients and controls on concrete, abstract neutral and emotional words. AD did not differ from controls in any of these categories, while the other two patients’ groups fared significantly worse: svPPA were significantly more impaired than all other groups with concrete and abstract neutral words, and both svPPA and bvFTD were impaired with emotion words. No groups showed any difference between performance on positive and negative emotion words.

In [51], svPPA and AD patients were tested with the same tests for abstract concepts from the DeCAbs battery [66], where stimuli belonged to five abstract categories, namely emotions, cognitions, traits, social relations, and human actions. While they were impaired in all the other categories, AD showed normal performance on two out of three tests with emotions (association task and sentence completion), while svPPA patients were selectively impaired in social relation concepts.

In another study testing both svPPA and AD [20], a similar performance was observed in AD patients for concrete, emotional abstract, and non-emotional abstract triplets in a similarity judgement task. SvPPA were better with abstract non-emotional than with concrete words, while performance on emotion triplets was intermediate. Ref. [33] svPPA case report, using a word-picture matching task, showed a non-significant better performance in a word-picture matching of abstract non-emotional compared to concrete and emotional abstract words, a pattern opposite to that of matched controls. In verbal fluency of abstract words, a svPPA patient [34] produced positive and negative feeling words (emotion) in the same number as controls, while she was impaired in concrete categories. Ref. [54] compared the performance of two controls with two svPPA with bilateral ATL atrophy, with respectively a predominant left or right atrophy, on a synonym judgement task on social concepts (abstract) and non-social concepts (properties of animals). The two patients were significantly impaired in both conditions, but the right ATL patient was significantly more impaired than the left on social concepts. An additional svPPA patient [22] showed a similar specific impairment for social concepts, compared to emotion and quantity concepts (abstract categories), animals and tools (concrete categories) on a lexical decision task: the patient showed abolished priming only for social concepts, while controls were primed in all conditions.

An overall summary of the results can be found in Table 2.

## 4. Discussion

In this scoping review, we aimed to identify the extent of evidence regarding abstract and concrete concepts knowledge in FTD (with a specific focus on svPPA) and AD patients and the related sites of atrophy. We also analyzed the difference concerning grammatical classes and, when available, semantic categories.

### 4.1. Abstract/Concrete Concepts Contrast and Related Site of Atrophy

According to the collected evidence, the reversal of the CE (A > C) appears as the most frequent pattern in svPPA patients. Most studies (14 out of 24) reported better performance with abstract compared to concrete concepts in svPPA, showing that in the majority of svPPA patients, the reversal of CE is present. This pattern differs from that of bvFTD patients, whose performance suggests an increase in the CE. It also deviates from that of AD, who, like bvFTD patients, showed more frequently a better performance with concrete over abstract concepts, with a significant change from this pattern when emotion concepts were included.

However, not all svPPA patients show the reversal of the CE: six records reported the opposite trend, with a better performance with concrete concepts, and the other four found a similar performance for abstract and concrete concepts. Taken together, these results are inconsistent and leave open the question as to why this reversal of CE appears. We will discuss possible explanations below, but first, some information about the neural substrates is required.

Some of the svPPA studies reported the ATL as the site of atrophy, predominantly on the left side. Inside the ATL, the reported areas of atrophy are heterogenous, including both ventral and superior portions of the ATL, as well as medial and lateral. The patients whose cortical atrophy was evaluated performed better on abstract concepts [33,34,35,36,37,38,39,40], better on concrete [44,52,54], or similarly on concrete and abstract concepts [55,58]. Five of these studies also reported a positive correlation between ATL atrophy and the reversal of the CE [20,36,37,38,39,40].

Different theories have been proposed to account for both patterns of performance in svPPA (A > C and C > A), ascribing both opposing roles to the same cortical region, namely the ATL.

According to the hub-and-spoke model, ATL represents a central, amodal hub where all conceptual knowledge is stored and represented. This hub receives inputs from different, modality-specific regions (the spokes) and combines them to create a unitary, coherent multi-modal conceptual representation [67,68]. According to this view, concepts are represented by means of the multi-modal representations (spokes) throughout the brain and by the central hub (ATL) that receives inputs from all of them. This theory has been put forward to explain svPPA cases that perform better on concrete than on abstract items (C > A). Since abstract concepts do not benefit from the same rich multisensory representation that supports concrete concepts [69], in the case of ATL atrophy, they shall be the first to a decade, whereas concrete concepts would still be supported by their richer representation. This theory is further supported by TMS evidence [42]: in this study, inhibitory stimulation on healthy subjects targeting ATL significantly slowed subjects’ semantic processing, particularly for less imageable (abstract) items. Neuroimaging studies [70,71] also highlight ventral and middle lateral ATL as the core regions for semantic processing.

To explain the reversal of the CE in svPPA (A > C), others observed how the ventral ATL (inferior temporal gyrus, fusiform gyrus, parahippocampal gyrus), the main target of svPPA atrophy [72], corresponds to high-level visual association areas [73,74]. Since these regions are specifically involved in concrete concepts (objects, animals) representation, concrete concepts would be primarily impaired, leading to the reversal of the CE observed in svPPA patients. In this review, five studies (see above) directly correlated the size of the reversal of the CE in svPPA patients to ATL atrophy. This connection is also supported by studies that found svPPA cases more impaired in living than in inanimate concepts [34,75,76] and more impaired in perceptual than functional features knowledge [33,35]. Indeed, according to the Sensory/Functional theory of category-specific disorders [5,77], living entities are defined mostly by their perceptual (visual) attributes, whereas inanimate ones are distinguished by both perceptual and non-perceptual (functional) features.

However, the ATL is not a unitary region: neuroimaging [21,78,79] and TMS [54] evidence showed how different portions of ATL are preferentially weighted towards the processing of abstract or concrete knowledge. [78]—an fMRI study—showed that there is a gradual specialization in ATL from dorsolateral to medial-ventral ATL: while the inferior temporal gyrus responds similarly to concrete and abstract concepts, superior and middle temporal gyri (dorsolateral) show a greater response to abstract, and fusiform and parahippocampal gyri (ventromedial) to concrete concepts. In another fMRI study on healthy participants [21], it was shown that bilateral superior ATLs are more strongly activated by social than animal concepts, and both types activate similarly middle ATL regions. In a following study on FTD and cortico-basal syndrome patients [79], the authors corroborated these results: patients with right superior ATL hypometabolism showed a selective impairment for social contrasted to animal concepts. Pobric et al. [54] used the same stimuli as [79] and repetitive TMS to inhibit right and left superior ATL: right superior ATL stimulation selectively impaired social concepts performance, while left stimulation impaired both social and animal concepts.

The gradual specialization of ATL might explain the seemingly controversial results in svPPA patients: the extent and precise location of grey matter atrophy differed across patients included in this review, and this might explain the opposite C > A and A > C patterns found in patients affected by the same disease. ATLs might preserve their central role as the semantic hub in the brain [67] while maintaining a graded specificity, whereby dorsolateral regions are preferentially involved in abstract concepts processing and ventromedial regions, corresponding to high-level visual association areas, are responsible for concrete concepts processing [74,78].

Another potential factor that could account for variability in svPPA semantic performance is the duration of the disease: the extent of atrophy increases with the progression of the disease, and so does the patients’ semantic impairment. Initial atrophy in svPPA is usually located in the left ATL, whereas a minority of patients present right ATL degradation first [80,81]. From that region, atrophy spreads posteriorly across the temporal lobe, involving visual association areas important for concrete concept representation and temporarily sparing inferior frontal regions more important for abstract concepts [82].

Patients tested at different time points from the beginning of the disease would consequently show different patterns of abstract/concrete concepts impairment. However, there are very few studies that investigated the longitudinal progression of semantic impairment in svPPA patients [35,38,83], and only one [38] directly assessed the connection between longitudinal changes in grey matter atrophy and semantic impairment. Ref. [83] found that concrete concepts strongly associated with visual experiences in svPPA patients become more impaired with the progression of the disease. In a longitudinal case report [35], the patient was tested on both concrete and abstract concepts at three time points: the patient showed an early reversal of the CE, which decreased with the progression of the disease. Ref. [38] tested a group of svPPA in the Cookie Theft picture description task [46] at two time points. They found a decrease in the concreteness of produced nouns with the progression of the disease, and this effect correlated with progressive grey matter atrophy in the left superior temporal gyrus and right ventral temporal regions. They also tested another group of bvFTD patients, which did not show any longitudinal effect of concreteness of produced nouns.

Five records also included bvFTD patients, and all reported better performance with concrete or similar performance with concrete and abstract concepts.

Four of them reported the related site of atrophy, which always included predominantly inferior frontal lobe regions, and to a lesser extent, temporal regions [36,37,39,58]. This is consistent with the typical extent of atrophy in this disease, which includes the dorsolateral, inferior, and orbital regions of the frontal lobe [36]. Ref. [36] also found that bilateral inferior frontal and insula atrophy correlated with the degree of concreteness effect in these patients. Taken together, this evidence is in line with neuroimaging studies showing a preferential involvement of the IFG in abstract concept processing [26,74].

Another aspect that emerged in this review is the distinction among grammatical classes.

### 4.2. Grammatical Classes: Nouns, Verbs, Adjectives

Two grammatical classes were investigated: nouns and verbs. A third class, adjectives, was evaluated only in two studies [34,55], and no difference between concrete and abstract items was revealed. The CE across grammatical classes is once again inconsistent. Some studies that tested the same patients in both classes reported an effect of concreteness only in nouns but not in verbs (i.e., the effect of concreteness specific to nouns, see for example [34], AD patients in [50]), only in verbs but not in nouns (i.e., the effect of concreteness specific to verbs, see svPPA patients in [47,50]). Other studies instead found no difference between grammatical classes [53]. There are no specific data on the atrophy in these patients. We can only refer to neuroimaging studies on healthy subjects, showing that motion and cognition verbs are represented in distinct regions [84,85]. In [84], motion verbs activated more anterior prefrontal regions and the temporal –occipital cortex, while cognition verbs recruited left posterior portions of the temporal cortex. Motion verb impairment was also correlated to bilateral prefrontal and motor association cortex atrophy in patients affected by amyotrophic lateral sclerosis [86]. Instead, another fMRI study contrasted concrete (motion) and abstract (including emotion, e.g., ‘love’) verbs [85] and found that the abstract verbs elicited higher activity in the bilateral IFG and anterior middle temporal lobe.

Given the discrepancies in the CE across nouns and verbs, and the likely differences in nouns and verbs cortical representation, future studies should investigate the effect separately for each grammatical class.

Finally, in studies showing a reversal of the CE, an association with a disproportionate impairment of living things has been described, and different results were found when considering different types of abstract concepts.

### 4.3. Semantic Categories

- Living/Non-living

Among the five studies that distinguished the living (biological)/non-living (inanimate) concrete categories in svPPA, two [33,35] found better processing of functional over perceptual features, and two [33,34] better knowledge of non-living compared to living entities; in all these patients, these effects appeared together with a reversal of the CE. The other two records [22,51] did not find evidence of category or features specific effects in svPPA; only [51] found better processing of non-living concepts in AD patients.

- Abstract categories

The included studies revealed a selective sparing of emotional concepts in AD patients and a selective impairment of social concepts in svPPA patients.

Despite the fact that AD patients’ performance most frequently reflects the CE found also in healthy subjects, when the abstract material consists of emotional words contrasted with concrete [20,51,58,63,64], or the relationship between concepts is emotional rather than neutral [62], the advantage shifts toward emotion concepts, resulting in no difference or in a reversal of the CE. These results are in line with the notion that affective processes are relatively preserved in the initial stages of AD and can thus facilitate the processing of emotional concepts [10].

In svPPA, the pattern with emotion words is more heterogenous, with reports of worse performance in emotion compared to other abstract concepts [33], similar performance between emotion and non-emotion abstract concepts [20,58], or preservation of emotion words over other categories of abstract concepts, like social ones [51]. In a neuroimaging and behavioral study [87], the authors also found impaired emotion concepts knowledge in svPPA patients, which was significantly correlated with emotion recognition; in turn, both measures correlated with grey matter atrophy in ventral frontal, temporal and insular regions.

Social concepts are found to be consistently more impaired when compared to other categories of abstract words in svPPA [22,51,54] or to concrete words [54,79].

Recent works suggested that, like the concrete, the abstract domain can be distinguished into different semantic categories, namely emotion, social, quantity, and theory of mind concepts. In line with an embodied view of abstract concepts cognition, these categories are grounded in the same distinct neural basis representing the corresponding experiences [19,88]. Likewise, multidimensional scaling studies showed that the concrete/abstract distinction is too simplified, and different dimensions/experiences organize the semantic space [89] into different concrete and abstract categories [90].

Taken together, the included studies also support the existence of different categories of abstract concepts that can be selectively impaired in patients affected by AD and svPPA.

This scoping review has some limitations. First, we analyzed together studies that used both concreteness and imageability ratings to classify a word as either concrete/abstract without distinguishing results based on the dimension used. However, it is known that emotional words are rated as more imageable than other abstract concepts but less concrete [91,92]. Even though the two measures are highly correlated [27], they are not synonymous and affect semantic performance differently [28,29,30,31].

Second, we did not distinguish results based on the type of task. We included studies assessing comprehension (mostly synonym judgement tasks) but also production (picture naming, oral descriptions of pictures) and semantic priming tasks. We cannot exclude that the effect of concreteness might vary depending on the type of task.

Third, we also included studies without a control group. In these studies, it is impossible to determine whether the difference in patients’ performance between abstract and concrete concepts reflects the same or different trend and/or to the same degree that a healthy control group would show.

## 5. Conclusions

The main aim was to assess the contrast between concrete and abstract concepts in svPPA and AD patients to shed light on the anatomical correlates of the reversed CE.

With regards to AD, the most frequent pattern was a better performance with concrete compared to abstract concepts. These patients also showed selective preservation of emotional abstract concepts, which were processed better than concrete and abstract neutral concepts.

Most svPPA showed a reversal of the CE, but a few studies also found the opposite trend or no difference between concrete and abstract. All svPPA patients presented with ATL atrophy, which in some cases also correlated with the size of the reversal of CE. We argue that to account for the discrepancies in svPPA performance across the concrete and abstract domain, two main factors must be considered. First, the ATL is not homogeneous, whereby the dorsolateral region responds more strongly to abstract, and the ventromedial region more to concrete concepts. Differences in size and location of atrophy in svPPA patients would consequentially give rise to opposite effects. Second, and related to the first point, the duration of the disease is a variable to control for, as longitudinal studies found the degree of reversal of the CE to change with the progression of the disease, along with the spread of the atrophy over the temporal lobe.

We also found that grammatical class influences the effects of concreteness, although in an inconsistent way, with effects specific to nouns, specific to verbs, or generalized across the two.

Finally, beyond the distinction of concepts across the concreteness spectrum, different semantic categories (emotion, social concepts for the abstract, living, non-living for the concrete domain) appear to be selectively impaired in svPPA and AD patients, suggesting that the concrete/abstract distinction is insufficient and a finer, multidimensional method is needed to characterize the neurodegenerative patients’ semantic impairment.

## Figures and Tables

**Table 1:** Results of concrete/abstract contrast for each disease. C > A: Concreteness effect, A > C: Reversal of concreteness effect, C = A: no difference between concrete and abstract concepts.

	SvPPA	BvFTD	NfPPA	LPPA	AD
C > A	6 records, 99 patients	3 records, 66 patients	3 records, 63 patients	2 records, 113 patients	5 records, 106 patients
A > C	14 records, 147 patients	/	/	/	2 records, 39 patients
C = A	4 records, 30 patients	2 records, 82 patients	/	/	4 records, 58 patients

**Table 2:** Summary of the 31 studies included in this review. Abbreviations: IFG: Inferior frontal gyrus, FG: fusiform gyrus, ITG: inferior temporal gyrus, ATL: anterior temporal lobes, n.a.: not available, n.s.: not specified, AD: Alzheimer’s disease, PPA: Primary Progressive Aphasia, SvPPA: semantic variant of Primary Progressive Aphasia, nfPPA: nonfluent variant of Primary Progressive Aphasia, lPPA: logopenic variant Primary Progressive Aphasia, uPPA: unclassified Primary Progressive Aphasia bvFTD: behavioral-variant Frontotemporal Dementia, CBS: Cortico-Basal Syndrome, C > A: Concreteness effect, A > C: Reversal of concreteness effect, C = A: no significant difference between concrete and abstract concepts.

Reference ID	Patients Type	Site of Atrophy	Semantic Categories	Grammatical Class	Short Results
[20]	AD, svPPA, controls	n.a.	Concrete, Abstract: emotion, non-emotion	n.s.	AD: C = A svPPA: A non-emotion > C controls: A emotion >C
[22]	CBS, svPPA, controls	n.a.	Concrete: living, non-living Abstract: emotion, social, quantity	Nouns	CBS: C > A quantity svPPA: C > A quantity controls: C = A
[33]	svPPA, controls	ATL, particularly left ATL	Concrete: living, non-living Abstract: non-emotion, emotion	Nouns, Verbs	svPPA: A > C controls: C > A
[34]	svPPA, controls	Left ATL	Concrete: living, non-living Abstract: non-emotion, emotion	Nouns, Verbs, Adjectives	svPPA: A > C Non-living > living controls: C = A
[35]	svPPA, Controls	ATL, particularly left ATL	Concrete: living, non-living	n.s.	svPPA: A > C
[36]	bvFTD, svPPA, controls	svPPA: ATL, particularly left ATL bvFTD: frontal lobes	Concrete, Abstract	Nouns	svPPA: A > C bvFTD: C > A controls: C = A
[37]	bvFTD, svPPA, controls	svPPA: left IFG, left FG, right ITG bvFTD: frontal lobes	Concrete, Abstract	Nouns	svPPA: A > C bvFTD: C > A
[38]	bvFTD, svPPA, controls	SvPPA: medial and lateral temporal regions	Concrete, Abstract	Nouns	svPPA: A > C bvFTD: C > A
[39]	bvFTD, nfPPA, svPPA, controls	SvPPA: ATL and orbitofrontal cortex NfPPA: left middle frontal, inferior and middle temporal regions BvFTD: frontal and temporal	Concrete, Abstract	Nouns	svPPA: A > C BvFTD, nfPPA, controls: C > A
[40]	svPPA, controls	ATL	Concrete, Abstract	Verbs	svPPA: A > C controls: C > A
[41]	svPPA, controls	n.a.	Concrete, Abstract	n.s.	svPPA: C > A controls: C > A
[44]	lPPA, nfPPA, svPPA, uPPA	svPPA: left anterior and inferior temporal lPPA: left temporo-parietal nfPPA: asymmetric frontal	Concrete, Abstract	Verbs	lPPA, nfPPA, svPPA, uPPA: C > A
[47]	svPPA	ATL, particularly left ATL	Concrete, Abstract	Nouns, Verbs	svPPA: A > C
[48]	svPPA, controls	n.a.	Concrete, Abstract	n.s.	svPPA: A > C controls: C > A
[49]	svPPA, controls	n.a.	Concrete, Abstract	n.s.	svPPA: A > C controls: C > A
[50]	AD, svPPA, controls	n.a.	Concrete, Abstract	Nouns, Verbs	AD: C > A (specific to nouns) svPPA: A > C (specific to verbs)
[51]	AD, svPPA, controls	n.a.	Concrete: living, non-living Abstract: Emotions, Cognitions, Traits, Social relations, Human actions	n.s.	AD: C = A svPPA: A > C
[52]	svPPA	ATL	Concrete, Abstract	Nouns, Verbs	svPPA: C > A
[53]	svPPA, controls	n.a.	Concrete, Abstract	Nouns, Verbs	svPPA: C > A
[54]	svPPA, controls	ATL, predominantly on the left or right side	Concrete: properties of animals Abstract: social	n.s.	svPPA: C > A
[55]	svPPA, controls	ATL	Concrete: colour, dimension, physical properties Abstract: human propensity, value	Adjectives	svPPA: C = A
[56]	bvFTD, lPPA, nfPPA, svPPA, controls	n.a.	Concrete, Abstract	n.s.	bvFTD, lPPA, nfPPA: C > A svPPA: C = A
[57]	AD, svPPA, controls	n.a.	Concrete, Abstract	n.s.	AD: C = A svPPA: C = A
[58]	AD, bvFTD, svPPA, controls	svPPA: ATL predominantly on the left bvFTD: frontal regions	Concrete, Abstract: emotion, non-emotion	n.s.	AD: C = A svPPA: C = A controls: C = A
[59]	AD, controls	n.a.	Concrete, Abstract	n.s.	AD: C > A, controls: C > A
[60]	AD, old controls, young controls	n.a.	Concrete, Abstract	Nouns, Verbs	AD: C > A young controls: C > A old controls: C = A
[61]	AD, old controls, young controls	n.a.	Concrete, Abstract	n.s.	AD: C > A old controls: C > A young controls: C > A
[62]	AD, controls	n.a.	Concrete: emotion and neutral Abstract: emotion and neutral	n.s.	AD: C neutral > A neutral
[63]	AD, controls	n.a.	Concrete, Abstract: emotion	n.s.	AD: A > C
[64]	AD, old controls, young controls	n.a.	Concrete, Abstract: emotion negative, emotion positive	n.s.	AD: A emotion negative > C and emotion positive Old, young controls: A emotion negative = A emotion positive
[65]	AD, PPA	n.a.	Concrete, Abstract	n.s.	Unclassifiable

## Data Availability

Not applicable.

## Supplementary Materials

The following supporting information can be downloaded at: https://www.mdpi.com/article/10.3390/brainsci13050765/s1, Table S1: Records with Primary Progressive Aphasia patients; Table S2: Records with Alzheimer’s disease patients; Table S3: Records with both Alzheimer’s disease and Primary Progressive Aphasia patients.
