# Supplementary material for: Concrete and Abstract Concepts in Primary Progressive Aphasia and Alzheimer’s Disease: A Scoping Review
Source: Brain Sci. 2023 May 5;13(5):765. doi: 10.3390/brainsci13050765 (PMC10216362; doi:10.3390/brainsci13050765)
Supplement: Supplementary file 1 [file brainsci-13-00765-s001.zip › brainsci-2368536-supplementary.pdf]

Table S1. Records with Primary Progressive Aphasia patients

REFERENCE	PARTICIPANTS	LESION	TASK	STIMULI/<br>MATERIAL	RESULTS
Breedin et al., 1994	1 svPPA patient, DM.<br>SINGLE CASE.<br>10 controls, matched in age to DM, in the Auditory lexical decision test	Atrophy of ATL, particularly on the left side	Abstract/Concrete contrast:<br>**1.** Defining Abstract and Concrete Words<br>**2.** Concrete/Abstract Word/Picture Matching<br>**3.** Abstract-Concrete Synonymy Task<br>**4.** Auditory Lexical Decision Test<br>**5.** Verb-Noun Synonymy Task<br>Perceptual components of concepts:<br>**6.** Living non-living attribute test<br>**7.** Perceptual features of verbs	**1.** 386 words, controlled for concreteness and frequency.<br>**2.** 30 abstract words, 30 concrete words, 15 emotion words<br>**3.** 52 synonymy triplets, half concrete and half abstract nouns<br>**4.** 360 stimuli, half words (abstract and concrete) and half non-words<br>**5.** 16 verbs triplets and 16 noun triplets<br>**6.** 39 living and 39 non-living items<br>**7.** 27 triplets of verbs per conditions: non-relational, manner, and relational triplets	**A>C**<br><br>Abstract/Concrete contrast:<br>DM was better in defining abstract compared to concrete words, and better in picture matching of abstract compared to concrete words, although the difference was not statistically significant.<br>Also, there was no difference in performance on synonymy judgement tasks between abstract/concrete triplets and between noun/verbs triplets.<br>In the auditory lexical decision test, similar performance on concrete and abstract words.<br><br>Perceptual components of concepts:<br>Worse with perceptual than non-perceptual features. Worse with manner triplets compared to the other two conditions
Reilly et al., 2007	4 svPPA<br>No control group	n.a.	**-** Concreteness judgement	**-** 40 nouns and 40 verbs, divided into concrete and abstract	**A>C**<br><br>Patients showed overall higher accuracy for three-syllable abstract, compared to concrete items
Macoir et al., 2008	SC, 1 svPPA patient<br>5 controls matched for age and education	Infero-lateral ATL atrophy, particularly on left side	Longitudinal study: T1, T2, T3<br>Abstract and concrete concepts knowledge. 5 tasks:<br>1. Semantic similarity judgement on homophones,<br>2. Semantic similarity judgement on living, non-living, and abstract concepts,<br>3. Concrete/abstract word-to-picture matching,<br>4. Concrete/abstract word definition,	1.32 noun homophones with both an abstract and a concrete meaning, from which 64 triplets, half with semantic similarity judgement of the concrete and the other half of the abstract meaning<br>2.20 concrete living, 20 concrete non-living, and 40 abstract triplets.	**A>C**<br><br>Contrast abstract/concrete items reveals a strong reversal of CE: SC performed better on abstract compared to concrete items. This difference progressively diminishes as the disease evolves.<br>No difference in performance between living and non-living entities

			5. Concrete/abstract word spelling to dictation<br>Living and non-living concrete items.<br>6. Picture naming<br>7. Naming to definition	3.40 stimuli of concrete concepts (20 living, 20 non-living), 40 stimuli of abstract concepts<br>4.43 concrete (living and non-living) and 47 abstract words<br>5. 45 concrete (19 living and 26 non-living) and 45 abstract word stimuli<br>6. 127 stimuli.<br>The living set: 61 pictures of animals (38) and fruits and vegetables (23);<br>the non-living set: 66 pictures of vehicles (7), tools (8), articles of clothing<br>(11), musical instruments (8) and household items (32).<br>7.same as in 6<br>-	
Bonner et al., 2009	11 svPPA,<br>16 controls, age- and education-matched	MRI on a subset of 5 patients: bilateral, infero-lateral ATL atrophy	Verb similarity test	20 concrete verbs,<br>20 abstract verbs	**A>C**<br><br>Controls: CE<br>svPPA: reversal of CE (worse performance with concrete than with abstract verbs).<br>The size of the reversal of CE increases with right anterolateral temporal cortex atrophy
Jefferies et al., 2009	11 svPPA,<br>11 controls, matched for age	n.a.	Synonymy judgement task	Concrete and abstract, with one probe, one target and two distractors	**C>A**<br><br>SvPPA performed better on more imageable trials.<br>No relationship between the degree of semantic impairment and the size of the CE
Papagno et al., 2009	1 svPPA<br>5 controls, sex, age and education matched	Hypodensity in the left temporal pole and medial temporal cortex	Concrete nouns:<br>1. proper names<br>2. living and inanimate objects<br>(i) a reality judgement task; (ii) a picture naming task; (iii) a semantic memory questionnaire<br>3. knowledge of visual features:<br>(i) Size comparison test<br>(ii) animal tails task	1.50 pictures of celebrities, interspersed with 50 pictures of unknown people.<br>2.30 stimuli of living entities, 30 stimuli of inanimate objects<br>3.(i) 16 pairs of items<br>(ii) 8 pairs of animals<br>(iii) 60 stimuli, 40 biological and 20 inanimate	**A>C**<br><br>Concrete nouns:<br>Impaired performance in naming proper names of conspecifics and landmarks.<br>Significant greater difficulty with naming animals compared to inanimate objects and significant loss of semantic knowledge for biological, whereas at ceiling with inanimate objects.

			(iii) colour attribution task<br>Abstract and concrete words:<br>4. Naming concrete and abstract nouns from definition<br>5. Word fluency<br>6. Synonymy task<br>7. Word-definition verification task	4.38 definitions (28 of concrete and 10 of abstract words)<br>5. categories: concrete animate (wild animals, pets, insects, citrus fruits, green vegetables) and inanimate (pieces of furniture, vehicles, tools), and abstract (positive and negative feelings)<br>6. sublist A: triplets of abstract and concrete nouns (26 each)<br>sublist B: triplets of nouns (16), verbs (16), and adjectives (40)<br>sublist C:3 groups of verbs triplets: manner triplets, opposite triplets, relational triplets (27 each)<br>7.236 definitions of words: 28 concrete and 52 abstract nouns, to 41 concrete and 47 abstract verbs, and to 30 concrete and 37 abstract adjectives	Knowledge of visual features is preserved for artefacts, but not for biological entities.<br>Abstract and concrete words:<br>Perfect naming for abstract concepts, impaired in naming concrete concepts (reversal of CE).<br>Fluency comparable to controls for abstract concepts, and impaired for concrete concepts (reversal of CE)<br>In sublist A, patients were significantly worse than controls in concrete, but not in abstract triplets (reversal of CE). In sublist B, performance gradually better from (lower to higher) nouns, adjectives, and verbs. In sublist C, performance is indistinguishable from controls.<br>In the word-definition verification task, better performance with abstract compared to concrete concepts, but only with nouns (reversal of CE, specific to nouns)
Hoffman and Ralph, 2011	7 svPPA,<br>No control group	Bilateral ATL atrophy in all cases. 4 patients present stronger atrophy on the left, 3 on the right side	7 tasks:<br>1. Synonym Judgment Task, (Jefferies et al., 2009)<br>2. Description-to-Noun Matching Task (Yi et al., 2007)<br>3. Description-to-Verb Matching Task, (Yi et al., 2007)<br>4. Verb Similarity Test, (Bonner et al., 2009)<br>5. Shallice and McGill (Unpublished Data) Word--Picture Matching Task,<br>6. Mischievous Monkey Test with Pictures<br>7. Mischievous Monkey Test with Words	1. 64 trials (56 nouns, 5 adjectives, 3 verbs). 3 levels of imageability<br>2. 20 concrete and 20 abstract nouns<br>3. 20 verbs of motion and 20 verbs of cognition<br>4. 40 trials, 20 concrete and 20 abstract<br>5. 30 abstract and 30 concrete trials<br>6. 48 concrete and 48 abstract words<br>7. Same as 6.	**C>A**<br><br>Overall, better performance with concrete than with abstract items.<br>Size of CE varies between tasks: stronger on synonymy judgement task, no effect on tasks probing verbs.

Hoffman et al., 2013	6 svPPA<br>10 controls, matched for age and educational level	n.a.	Experiment 1.<br>Synonymy matching task<br>Experiment 2.<br>Associative relationship task, with a probe word and three choices	Experiment 1.<br>60 nouns and 60 verbs, half concrete and half abstract<br>Experiment 2.<br>3 conditions:<br>1. 40 trials with abstract words with shared associative relationship<br>2. 40 trials with concrete words shared associative relationship, not perceptually similar<br>8. 40 trials with the same concrete probes, associative relationship, and also perceptually similar	**C>A**<br><br>Experiment 1.<br>SvPPA showed larger CE than controls, for both word classes<br><br>Experiment 2.<br>SvPPA performed more poorly in abstract condition, compared to both concrete conditions, and better on perceptual concrete compared to the associative concrete condition
Hoffman et al., 2014	7 svPPA,<br>8 controls, age- and education-matched	n.a.	Autobiographical memory interview.<br>Frequency, imageability and semantic diversity of words produced obtained		**A>C**<br><br>SvPPA produced more high-frequency and high semantic diversity words than controls and less high-imageable words
Macoir et al., 2015	4 svPPA patients,<br>12 controls	Bilateral ATL atrophy	Experiment 1.<br>1. Semantic judgment tasks about concrete and abstract adjectives<br>2. Semantic knowledge task about colour adjectives<br>Experiment 2<br>1. Adjective-to-concrete noun matching task:	Experiment 1.<br>Adjectives of concrete (colour, dimension, physical property) and abstract (human propensity, value) semantic types.<br>Experiment 1.<br>1. 50 triplets for the synonymy judgement task, and 50 triplets the for antonymy judgement task. 25 concrete, 25 abstract<br>Experiment 2.<br>1. Same adjectives used in experiment 1. 80 stimuli comprising the	**C=A**<br><br>SvPPA performed significantly below controls in all tasks and semantic categories. No significant difference across categories in patients' performance (no effect of concreteness)

				adjective and three object nouns	
Woollams, 2015	10 svPPA,<br>30 healthy (not matched)	n.a.	Reading aloud	80 monosyllabic words, varied by imageability and consistency	**A>C**<br>In controls, faster RTs for inconsistent high vs low imageability items, in svPPA, reversal: faster RTs for inconsistent low vs high imageability items
Cousins et al., 2016	12 svPPA,<br>18 bvFTD,<br>18 controls	SvPPA: cortical atrophy of temporal lobes, particularly on the left hemisphere.<br>BvFTD: frontal lobe regions atrophy	Associativity judgement task	60 triads of nouns, half concrete and half abstract.	**A>C**<br><br>Controls: no abstract-concrete difference<br>SvPPA: reversal of CE, i.e. higher accuracy for abstract compared to concrete triads<br>BvFTD: CE, i.e. higher accuracy for concrete than abstract triads.<br>In svPPA, reversal of CE is related to atrophy of the left anterior temporal cortex
Pobric et al., 2016	2 svPPA patients<br>30 controls	One patient with predominant right superior ATL atrophy, and the other with predominant left superior ATL atrophy	Synonym judgement task	Triplets of words, with two conditions: social concepts and non-social concepts (describing non-social behaviour or properties of animals)	**C>A**<br><br>Both cases were impaired compared to the control group in both conditions.<br>Social concepts were significantly more impaired compared to non-social concepts in the right ATL than in the left ATL patient.
Cousins et al., 2017	20 svPPA,<br>42 bvFTD,<br>32 controls	SvPPA: cortical atrophy of left IFG, left FG, and right ITG.<br>BvFTD: atrophy of frontal lobe regions, also extended to temporal lobes	Cookie theft picture description task.<br>The abstractness of transcribed descriptions (nouns) was rated		**A>C**<br><br>SvPPA produced significantly more abstract nouns than bvFTD.<br>SvPPA more impaired in other measures of semantic knowledge also produced more abstract nouns than those less impaired.<br>Increased abstractness in svPPA related to atrophy in PHG and portions of left ATL
Cousins et al., 2018	11 svPPA,<br>15 bvFTD<br>No control group	SvPPA: grey matter atrophy in medial and lateral temporal regions, and inferior frontal lobe	Longitudinal study: T1 and T2 Cookie Theft Picture description task.<br>The abstractness of transcribed descriptions (nouns) was rated		**A>C**<br><br>Longitudinal decrease of concreteness of produced nouns in svPPA, but not in bvFTD.<br>The decrease of concreteness in svPPA was related to progressive atrophythe of right ventral and left superior temporal regions

Cho et al., 2021	138 FTD:<br>42 svPPA,<br>22 nfPPA,<br>74 bvFTD<br>37 controls	SvPPA: cortical thinning in ATL and orbitofrontal cortex, particularly on the left hemisphere.<br>NfPPA left middle frontal, inferior temporal and middle temporal regions.<br>BvFTD cortical thinning in frontal and temporal lobes bilaterally	Cookie Theft Picture description task.<br>Abstractness and other lexical variables of transcribed descriptions (nouns) were rated		**A>C**<br><br>SvPPA produced more abstract nouns compared to bvFTD, nfPPA and controls.<br>In svPPA, an increase the in the abstractness of produced nouns correlated with cortical thinning in left anterior temporal regions
Catricalà et al., 2021	2 patients:<br>P01, svPPA patients<br>P02, CBS patient<br>8 controls	n.a.	Lexical decision task, with a semantic priming paradigm	120 nouns, 120 pairs.<br>Conditions: word-pseudowords pairs, word-word pairs of the SAME category, word-word pairs of DIFFERENT categories<br>Concrete categories: animals, tools<br>Abstract categories: emotion, social, quantity	**C>A**<br><br>Controls showed a priming effect for all categories. P01 showed abolished priming for social pairs. P02 showed abolished priming for quantity-related concepts
Poos et al., 2022	FTD diagnosed:<br>9 svPPA,<br>10 lPPA,<br>9 nfPPA,<br>6 bvFTD<br>FTD genetically risk:<br>10 bvFTD,<br>1 nfPPA<br><br>59 controls, matched for sex, age and education	n.a.	- Test Relaties Abstracte Concepten (TRACE). One probe word, one target and three distractors<br><br>- Semantic Association Test (SAT): concrete counterpart of TRACE. One probe, one target, three distractors	- 30 items, abstract concepts<br>- 30 items, concrete concepts	**C=A**<br><br>Difference between TRACE and SAT (abstract and concrete concepts knowledge): bvFTD, nfPPA, lPPA were significantly worse on TRACE than on SAT, while svPPA were equally impaired (i.e., no CE in svPPA)

Stockbridge et al., 2022	72 SvPPA,<br>103 lPPA,<br>63 nfPPA,<br>29 uPPA	svPPA: left anterior and inferior temporal atrophy<br>lPPA: left temporo-parietal atrophy<br>nfPPA: asymmetric frontal atrophy	Hopkins Action Naming Assessment (HANA), a verb picture naming tasks	30-item verb pictures	**C>A**<br><br>Similar CE for lPPA, svPPA and unclassified PPA: increased verb concreteness related to better performance

Abbreviations. IFG: Inferior frontal gyrus, FG: fusiform gyrus, ITG: inferior temporal gyrus, ATL: anterior temporal lobes, PHG: para-hippocampal gyrus, n.a.: not available, CE: Concreteness effect, SvPPA: semantic variant of primary progressive aphasia, nfPPA: nonfluent variant of Primary Progressive Aphasia, lPPA: logopenic variant primary progressive aphasia, bvFTD: behavioural-variant Frontotemporal dementia, CBS: Cortico-Basal Syndrome, C>A: Concreteness effect, A>C: Reversal of concreteness effect, C=A: no difference between concrete and abstract. The effects of concreteness reported in the abbreviations refer to svPPA patients.

Table S2. Records with Alzheimer's disease patients

REFERENCE	PARTICIPANTS	TASK	STIMULI/<br>MATERIAL	RESULTS
Martin and Fedio, 1983	14 AD,<br>11 controls, matched for age, sex and education	Word-finding tests:<br>1-Boston naming test,<br>2-Fluency test<br>Word meaning tests:<br>3-Pleasantness ratings.<br>4-Symbol referent test	1-85 line-drawings of objects<br>2- n.a.<br>3-10 pleasant words, 10 unpleasant words, 10 neutral words<br>4-42 cards: 9 objects, 15 actions, 9 emotion words, 9 modifiers	**A>C**<br><br>AD performed significantly worse than controls in the Boston naming test and generated fewer words than controls in the fluency test. There was no difference in performance in the pleasantness ratings. In the symbol referent test, AD performed worse than controls in objects, actions, and modifiers but not in emotions.
Rissenberg and Glanzer, 1987	Experiment 1.<br>14 AD,<br>31 young healthy,<br>31 old healthy<br>Experiment 2.<br>12 AD,<br>20 young,<br>15 old healthy	Experiment 1.<br>Free recall of word lists<br><br>Experiment 2.<br>Word-finding	Experiment 1.<br>5 16-item word lists of abstract and concrete words<br><br>Experiment 2.<br>Definitions of 44 concrete and abstract words	**C>A**<br><br>Experiment 1.<br>Typical strong CE in the young, and in the AD group, but no CE in the old healthy group.<br><br>Experiment 2.<br>Word-finding ability was impaired in AD but not in the old healthy group. AD showed strong CE:

				they were significantly more impaired with word finding for abstract compared to concrete items
Bushell and Martin, 1997	20 AD,<br>16 controls	Semantic priming paradigm	80 related stimulus pairs, 20 for each condition: motion verb pairs, non-motion verb pairs, concrete nouns, and abstract nouns.<br>+ Unrelated stimulus pairs	**C>A**<br><br>Neither controls nor AD showed semantic priming for abstract nouns and non-motion verbs.<br>While controls showed priming for both motion verbs and concrete nouns, AD showed priming only for concrete nouns.
Fleming et al., 2003	25 AD,<br>19 old healthy,<br>27 young healthy	Immediate recall	3 lists, 15-words lists for young, and 12 words lists for old and AD. Each word had positive, neutral, or negative connotations. Words were matched for concreteness	**A>C**<br><br>AD performed better on immediate recall of emotionally laden words than neutral words, and negative words were recalled more than positive or neutral
Peters et al., 2009	16 AD,<br>16 controls matched for age and sex,<br>16 young	- Immediate serial recall,<br>- Synonym judgement task	- Lists of high-imageable and low-imageable words<br>- Pairs of low- and high-imageable words	**C>A**<br><br>CE in both tasks: AD performed worse than controls and young only in the low-imageability lists, and were less accurate with low-imageable pairs, compared to controls and young
Giffard and al., 2015	15 AD,<br>31 controls	Semantic priming paradigm, with 4 semantic priming effects tested for:<br>Concrete-neutral SP,<br>Abstract neutral SP,<br>Concrete emotional SP,<br>Abstract emotional SP	576 pairs of stimuli, rated on concreteness and emotional valence<br>9 prime-target conditions:<br>1. Concrete semantic and emotional relationship<br>2. Abstract semantic and emotional relationship<br>3. Concrete semantic relationship<br>4. Abstract semantic relationship<br>5. Concrete no semantic relationship but emotional target<br>6. Abstract no semantic relationship but emotional target	**C>A**<br><br>In AD, in neutral conditions, CE: priming for concrete, no priming for abstract neutral concepts. In emotional conditions, no CE: similar priming effects for concrete negative and abstract negative SP conditions.

			7. Neutral concrete words<br>8. Neutral abstract word<br>9. Word-nonword pairs	

Abbreviations: AD: Alzheimer's disease, CE: concreteness effect, SP: semantic priming, C>A: Concreteness effect, A>C: Reversal of concreteness effect, C=A: no difference between concrete and abstract

Table S3. Records with both Alzheimer's disease and Primary Progressive Aphasia patients

REFERENCE	PARTICIPANTS	ATROPHY SITE	TASK	STIMULI/ MATERIAL	RESULTS
Westbury et al., 2002	11 AD,<br>11 PPA,<br>No controls group	n.a.	Psycholinguistic Assessment of Language (PAL) battery	n.a.	**UNCLASSIFIABLE**<br><br>No significant difference in naming tests. Large differences in abstract words comprehension: PPA were significantly more impaired than AD, in both auditory and written modalities
Crutch and Warrington, 2006	20 AD,<br>9 svPPA,<br>40 controls	n.a.	1. Synonym comprehension test<br>2. as in 1., with three conditions:<br>a. antonym distractor<br>b. distant distractor<br>c. close distractor	1. abstract and concrete words<br>2. 48 abstract words	**AD: C=A**<br>**svPPA: C=A**<br><br>1. svPPA achieved significantly lower scores than AD and controls. No difference between concrete and abstract concepts' performance<br>2. AD and svPPA showed a steeper rate of decline than controls from the more general to more specific synonym judgement condition
Yi et al., 2007	29 AD,<br>12 svPPA,<br>17 controls	n.a.	Multiple-choice naming-to-description task	40 verbs (motion and cognition)<br>40 nouns (20 concrete and 20 abstract)	**AD: C>A**<br>**svPPA: A>C**<br><br>AD: more impaired with abstract compared to concrete nouns, and with verbs than nouns. No

					significant difference between motion and cognition verbs<br>SvPPA: more impaired with verbs compared to nouns. No difference between concrete and abstract nouns. Reversal of CE with verbs: svPPA were more impaired with motion compared to cognition verbs
Hsieh et al., 2012	12 AD<br>16 FTD:<br>8 svPPA, 8 bvFTD<br>15 controls	svPPA: ATL atrophy, predominantly on the left<br>bvFTD: frontal atrophy	1-Graded Synonyms test<br>2-Emotion word tests<br>2.a. Emotion word synonyms test<br>2. b. Emotion word association test	1-concrete and abstract non-emotional words<br>2-80 emotion words, 40 positive and 40 negative	**AD: C=A**<br>**svPPA: C=A**<br><br>1 and 2.a.: svPPA were significantly more impaired than all other groups.<br>2.b.: both svPPA and bvFTD showed worse performance than the other groups.<br>No difference between positive and negative emotion words in all dementia and the control group.
Catricalà et al., 2014	14 AD,<br>6 svPPA,<br>20 controls	n.a.	1)For concrete concepts, three tests of the CaGi battery:<br>1-Picture naming task<br>2-naming on oral description<br>3-sentence verification of 480 features<br>2)For abstract concepts, Three tests from the DeCAbs battery<br>1-sentence completion task,<br>2-Multiple verbal choice matching,<br>3-Association task	1)48 stimuli, divided into 2 categories: living and non-living categories<br><br>2)same 40 stimuli divided into 5 categories:<br>Emotions,<br>Cognitions,<br>Traits,<br>Social relations,<br>Human actions	**AD: C=A**<br>**svPPA: A>C**<br><br>Reversal of CE in svPPA: patients showed better performance with abstract concepts, particularly in comprehension tasks<br>AD showed better performance with non-living than living entities in the naming on oral description task<br>AD show a normal performance on Emotion concepts in 2 tasks out of 3, while svPPA show impaired performance on Social Relations in the association task

Joubert et al., 2017	12 AD,<br>9 svPPA,<br>11 controls,<br>matched for age and education		Similarity judgement task	30 trials with 10 triplets for each condition:<br>1. concrete<br>2. emotional abstract<br>3. non-emotional abstract	**AD: C=A**<br>**svPPA: A>C**<br><br>SvPPA performed worse than AD and controls, and AD performed worse than controls.<br>In controls, better performance for emotion triplets; in AD, no word type effect, in svPPA, better performance with non-emotional abstract words compared to concrete, while the difference with emotional words is not significant.<br><br>Positive correlation between the semantic judgement of concrete triplets and GM volume in left ATL, medial and lateral

Abbreviations. AD: Alzheimer's disease, svPPA: semantic variant Primary Progressive Aphasia, bvFTD: behavioural-variant Frontotemporal dementia, n.a.: not available, GM = grey matter, ATL: Anterior Temporal Lobe, C>A: Concreteness effect, A>C: Reversal of concreteness effect, C=A: no difference between concrete and abstract
